# A Young Patient With Acute Ostial Right Coronary Artery Aneurysm Presenting As ST Elevation Myocardial Infarction

**DOI:** 10.7759/cureus.58063

**Published:** 2024-04-11

**Authors:** Zahid Khan

**Affiliations:** 1 Acute Medicine, Mid and South Essex NHS Foundation Trust, Southend-on-Sea, GBR; 2 Cardiology, Barts Heart Centre, London, GBR; 3 Cardiology and General Medicine, Barking, Havering and Redbridge University Hospitals NHS Trust, London, GBR; 4 Cardiology, Royal Free Hospital, London, GBR

**Keywords:** triple antithrombotic therapy, rivaroxaban dosing, st elevated myocardial infarction (stemi), glycoprotein iib/iiia inhibitors, right coronary artery aneurysm

## Abstract

A coronary artery aneurysm (CAA) is a localized dilatation of a coronary artery segment >1.5 times the diameter of the adjacent normal segment. CAA is more common in men than women and has multiple etiologies, including genetic causes, infections, and atherosclerotic diseases. Kawasaki disease is the most common cause of CAA in children, whereas atherosclerosis is the most common etiology in adults. We present the case of a male in his 30s who presented with sudden-onset chest pain and inferior ST segment elevation on an ECG. Echocardiography revealed preserved left ventricular function and mild hypokinesia. The patient underwent an emergency coronary angiogram that showed an ostial right CAA with thrombi. He was initially managed with a glycoprotein IIb/IIIa inhibitor tirofiban infusion, followed by triple therapy with aspirin, clopidogrel, and rivaroxaban. The patient underwent magnetic resonance imaging of his head, which was normal, and he did not attend outpatient computed tomography coronary angiography. The patient was discharged with lifelong rivaroxaban 20 mg once daily.

## Introduction

Coronary artery aneurysm (CAA) is a rare entity with a reported incidence of 0.3-5.3% and a mean incidence of 1.65% from pooled analysis [1]. CAA is defined as the localized dilatation of an artery with a diameter >1.5 times greater than that of a normal adjacent artery. Atherosclerosis is the most common cause of CAA in adults, whereas Kawasaki disease (KD) is the main cause in children [1,2]. Other etiologies of CAA include congenital or genetic causes, infections, connective tissue diseases, and inflammatory conditions [1,2]. CAA affects men more commonly than women. Thromboembolism is a known complication in CAA-predisposing patients with acute ST-elevated myocardial infarction (STEMI) due to vessel occlusion [1,3]. Thrombus formation is favored by a sluggish flow through a dilated aneurysmal vessel.

COVID-19 is associated with CAAs with thromboembolic phenomena [2,4]. Patients with CAA may present with angina, rupture, or STEMI. Thrombus formation occurs in these vessels due to reduced blood flow in the aneurysm, resulting in partial or complete occlusion of the flow distal to the aneurysm. KD is responsible for 20% of untreated cases in children, whereas 90% of adult cases are caused by atherosclerosis [4,5]. CAAs can affect any of the coronary arteries; however, the incidence of left main stem CAA affects only 0.1% of the population [6,7]. Managing patients presenting with STEMI secondary to CAA using primary coronary angioplasty can be challenging.

A study from the United States compared the mortality rates of patients with STEMI with and without CAA [8]. Younger patients with CAA in this group had a higher prevalence of KD, and most of these patients were treated with coronary artery bypass grafting (CABG), thrombectomy, and bare metal stent implantation. CAA is diagnosed using echocardiography in the acute and subacute phases, and both absolute arterial and z-scores are used as the diagnostic criteria for CAA. CAA requires close monitoring owing to the high risk of thrombosis, and these complications are likely to occur in the largest giant CAA [5]. We present the case of a 37-year-old patient with a right coronary artery ostial aneurysm with thrombus formation presenting as inferior STEMI who was initially treated with triple therapy followed by lifelong anticoagulation therapy.

## Case presentation

A 37-year-old male ectomorph patient presented with sudden-onset chest pain while having a shower. He described the pain as sharp and central, worsening with breathing, accompanied by cold, clammy skin, and sweating. He had no significant medical history, and his family history was unclear due to estrangement. The patient denied any previous diagnosis of KD. He was not on any regular medication and was a nonsmoker and nondrinker. His initial ECG with ambulance services showed inferior STEMI, and he was brought to our center for acute STEMI. He denied fever, cough, shortness of breath, or coryzal symptoms. He described the initial pain as 10 on a severity scale that had eased to 4/10 with morphine, aspirin, and glyceryl trinitrate sprays.

His vital signs were as follows: respiratory rate of 17 breaths per minute, SpO2 of 100% on room air, temperature of 36.4°C, and blood pressure of 129/79 mmHg. He weighed 70 kg, was 178 cm tall, and had a BMI of 22.09 kg/m². Bedside echocardiography demonstrated inferior wall hypokinesia with a preserved left ventricular ejection fraction. The patient underwent an emergency coronary angiogram that demonstrated a right coronary artery ostial aneurysm of about 8 mm in size with heavy thrombus formation and an occluded distal posterior descending artery (Videos 1-3). He was administered two boluses of a glycoprotein IIb/IIIa inhibitor (tirofiban), followed by a tirofiban infusion (12.5 mg) in a 250 mL solution at a rate of 0.15 micrograms/kg/minute for six hours. The patient commenced treatment with enoxaparin 70 mg twice daily, along with aspirin and clopidogrel 75 mg once daily (OD). He also received 300 mg of aspirin and 600 mg of clopidogrel. A departmental echocardiogram showed normal left ventricle (LV) wall thickness and mildly impaired LV systolic function with an estimated ejection fraction of 52% on Simpson’s biplane (Videos 4, 5).

**Video 1:** Coronary angiogram shows right coronary artery ostial aneurysm

**Video 2:** Coronary angiogram shows right coronary artery ostial aneurysm and distal posterior descending artery occlusion

**Video 3:** Coronary angiogram shows normal left-sided coronary arteries

**Video 4:** Parasternal long-axis echocardiography view shows normal left ventricular function

**Video 5:** Apical 4 chamber echocardiography view shows a normal LVEF LVEF, left ventricular ejection fraction

The laboratory test results are presented in Table 1.

**Table 1:** Lab test results for the patient during admission

Lab test	Day 1	Day 2	Day 3	Reference values
Hemoglobin	157	160	155	120-150 g/L
White cell count	16.2	13.7	10.4	4-10 × 10^9^/L
Platelets	408	521	441	150-410 × 10^9^/L
Neutrophil	14.4	10.8	7.1	2-7 × 10^9^/L
Urea	6	3.9	4.5	2.5-7.8 mmol/L
Creatinine	83	92	97	45-84 umol/L
Sodium	136	140	139	133-146 mmol/L
Potassium	4.4	5	4.9	3.5-5.3 mmol/L
C-reactive protein	1	13	10	0-5 mg/L
Troponin T	438	2,524	2,345	0-14 ng/L
Total cholesterol	5.6	-	5.5	0-5 mmol/L
Low-density lipoprotein cholesterol	3.4	-	3.4	<2.5 mmol/L

Echocardiography demonstrated regional wall motion abnormalities in the akinetic mid-inferior wall segments and the dyskinetic apical inferior segments. He was discussed in a cardiovascular multidisciplinary team meeting, and the recommendation was to continue clopidogrel 75 mg and rivaroxaban 20 mg OD. Autoimmune screening, including vasculitis screening, such as rheumatoid factor, perinuclear and cytoplasmic antineutrophil cytoplasmic antibodies, antinuclear antibodies, erythrocyte sedimentation rate, complement levels, immunoglobulins, and cryoglobulins, resulted in negative results, except for gastric parietal cell antibodies. Tests for viral hepatitis and HIV were negative. He was discharged home with clopidogrel 75 mg OD, rivaroxaban 20 mg OD, bisoprolol 1.25 mg OD, ramipril 2.5 mg OD, lansoprazole 30 mg OD, and atorvastatin 40 mg OD. He was advised to continue with clopidogrel and rivaroxaban for 12 months, followed by lifelong clopidogrel.

The patient underwent outpatient magnetic resonance angiography (MRA) of the kidneys and brain to exclude any vascular abnormalities. MRA did not reveal any evidence of intracranial aneurysms or arteriovenous malformations, and no features were suggestive of vasculitis. The patient was booked for outpatient computed tomography coronary angiography (CTCA); however, the patient did not attend the outpatient appointment for CTCA.

## Discussion

CAA was first described by Morgagni in 1761, and the term aneurysm was used to describe localized saccular or fusiform-shaped abnormal dilatation of a coronary artery. In contrast, ectasia is used to describe diffuse dilatation [7]. Another type of CCA known as “giant aneurysm” is used for dilatation of the coronary artery that exceeds the reference vessel diameter by at least four times [7]. CAA is predominantly caused by atherosclerosis in adults and by KD in children. It affects males more than females, which is likely due to the higher incidence of atherosclerosis in males [1,3]. Other causative factors for CAA include Takayasu arteritis, genetics, and coronary artery stent complications [2]. CAA is mostly an incidental finding, although it may occasionally present with angina and plaque rupture. Sluggish blood flow in the aneurysm can result in partial or complete occlusion of the blood flow distal to the aneurysm, and patients may present with STEMI in such scenarios [2].

KD is a common risk factor for CAA in the Japanese population [5,9]. Children with KD may present with persistent fever and clinical features including conjunctival injection, lymphadenopathy, exanthema, and changes in the mucosae and extremities [5,9]. KD treatment aims to prevent CAA complications. The incidence of KD in European children is approximately five to 10 per 100,000 children under five years of age, whereas a higher prevalence is reported in Asian countries, with the highest incidence reported in Japan [5,10-14]. The incidence of KD in Japan has been reported to be 265/100,000 in children under five years of age, and its incidence is likely to increase. The male-to-female ratio for KD is 1.5:1 in Japanese children [14,15].

Coronary angiography is valuable for assessing CAA, and intravascular ultrasound can help determine the luminal composition of aneurysms and differentiate between true and pseudoaneurysms [1,16]. There is a lack of consensus on the best treatment strategy for CAA, and the guidelines are unclear. However, medical management is preferred for patients with CAA and atherosclerotic coronary artery disease. The management of CAA should be based on individual cases. Generally, smaller aneurysms <10 mm are medically managed with dual antiplatelet therapy (DAPT) and anticoagulants, such as rivaroxaban, whereas larger aneurysms >10 mm are managed with percutaneous coronary intervention (PCI) [17]. Giant aneurysms mostly require surgical intervention [18]. The optimal medical therapy for the management of CAA includes antiplatelet medication, anticoagulation, and statins, particularly if thrombosis or emboli are a concern [1,18].

The Prevention of Bleeding in Patients with AF undergoing PCI (PIONEER AF-PCI) study demonstrated a lower bleeding risk when rivaroxaban was used in combination with DAPT compared with vitamin K antagonisms, such as warfarin [19,20]. Statins play a role in CAA, as inflammatory cytokines and matrix metalloproteinase have been linked to CAA [1]. Invasive options, such as PCI and surgery, should be based on individual cases. PCI, which includes stent and coil insertion, is usually reserved for smaller aneurysms, whereas surgical techniques with or without CABG are reserved for saccular aneurysms or aneurysms >10 mm in size [20]. Our patient was initially treated with a glycoprotein IIb/IIIa inhibitor, a tirofiban infusion, and DAPT, followed by DAPT and rivaroxaban. He was discussed in a multidisciplinary team meeting, and a consensus was reached for medical therapy given his low-risk bleeding profile and young age. We could not ascertain the family history or prior diagnosis of KD.

Our patient was treated medically because of his young age and an aneurysm size of approximately 12 mm. A previous study did not show any difference in terms of major adverse cardiovascular outcomes in patients with coronary aneurysms who underwent medical, surgical, or PCI [21]. There is an increase in stent restenosis in patients who undergo PCI, particularly those with a CAA size >10 mm. Szalat et al. reported that in older patients with CAA sizes ranging from 10 mm to 35 mm who underwent PCI, five out of 24 patients had stent restenosis on a follow-up coronary angiogram. Surgery is generally reserved for high-risk CAA with an increased risk of rupture in symptomatic patients [22,23].

## Conclusions

CAAs are rare and can be life-threatening. Patients may present with STEMI and acute coronary syndrome. The management of CAA should be based on individual cases, and there is no clear consensus regarding the best treatment strategy. Smaller CAAs are generally medically managed, whereas larger CAAs may require stenting and giant aneurysms may require surgery. Medical therapy preferably includes DAPT and anticoagulation with a nonvitamin K antagonist.
